# An unusual cause of metabolic alkalosis: hiding in plain sight

**DOI:** 10.1186/s12882-020-01967-7

**Published:** 2020-07-23

**Authors:** Carmen Elena Cervantes, Steven Menez, Bernard G. Jaar, Mohamad Hanouneh

**Affiliations:** 1 Department of Medicine, Division of Nephrology, The Johns Hopkins University School of Medicine, Baltimore, MD USA; 2 The Welch Center for Prevention, Epidemiology, and Clinical Research, Baltimore, MD USA; 3 Department of Epidemiology, The Johns Hopkins Bloomberg School of Public Health, Baltimore, MD USA; 4 Nephrology Center of Maryland, Baltimore, MD USA

**Keywords:** Metabolic alkalosis, Hypokalemia, Baking soda, Sodium bicarbonate, Toxicity

## Abstract

**Background:**

Sodium bicarbonate, in the form of baking soda, is widely used as a home remedy, and as an additive for personal and household cleaning products. Its toxicity has previously been reported following oral ingestion in the setting of dyspepsia. However, its use as a non-ingested agent, like a toothpaste additive, has not been reported as a potential cause of toxicity.

**Case presentation:**

We are reporting a case of an 80-year-old woman who presented with chronic metabolic alkalosis and hypokalemia secondary to exogenous alkali exposure from baking soda as a toothpaste additive, which might have represented an underreported ingestion of the substance.

**Conclusions:**

Considering that one teaspoon of baking soda provides approximately 59 m-equivalents (mEq) of bicarbonate, specific questioning on its general use should be pursued in similar cases of chloride resistant metabolic alkalosis.

## Background

Sodium bicarbonate is widely known for its multiple uses as a home remedy and as an additive for personal and household cleaning products. As a strong base, it serves as a buffer to neutralize acid, and its consumer use stems from its properties as a deodorizing, degreasing, and cleaning agent. Sodium bicarbonate toxicity manifesting as metabolic alkalosis has previously been reported following oral ingestion in the setting of dyspepsia [1, 2]. However, its use as a non-ingested agent, like a toothpaste additive, has not been reported as a potential cause of toxicity.

## Case presentation

An 80-year-old woman with a past medical history of long-standing hypertension leading to chronic kidney disease (CKD) stage G3bA1 (baseline serum creatinine 1.4–1.78 mg/dL [123.7–157.35 umol/L], eGFR 33–45 mL/min), hyperlipidemia, atrial fibrillation and gastroesophageal reflux disease was referred to the Nephrology clinic by her primary care provider for evaluation of chronic metabolic alkalosis.

During her initial clinic visit, the patient endorsed decreased appetite and poor oral intake with resultant 5-pound weight loss over the course of 6 weeks. She denied any nausea, vomiting or diarrhea. Her home medications included metoprolol tartrate 50 mg twice daily, pravastatin 10 mg daily, pantoprazole 40 mg daily, rivaroxaban 15 mg daily, and calcium carbonate 600 mg daily. She specifically denied taking any additional medications, either prescribed or over-the-counter. On physical examination, the patient had a BMI of 17.2 kg/m^2^, blood pressure of 160/80 mmHg and heart rate of 80 beats per minute. She was clinically euvolemic on exam, particularly, there was no peripheral edema. Table 1 summarizes the patient’s initial serum laboratory findings. Notably, her serum bicarbonate was elevated to 44 mmol/L, urinalysis was unremarkable, with no proteinuria or active urine sediment, and urine pH of 6.0, which was somewhat lower than expected for her degree of metabolic alkalosis. Based on the arterial blood gas result with a pH of 7,50, CO2 53 mmHg (7.06 kPa) and bicarbonate 41 mEq/L, the patient was diagnosed with a primary compensated metabolic alkalosis. Spot urine electrolytes revealed the following: urine sodium of 41 mmol/L (fractional excretion of 0.3%), potassium 13.0 mmol/L (fractional excretion 3.87%) and chloride 48 mmol/L (fractional excretion of 0.5%), with a urine creatinine of 128 mg/dL (11,317 umol/L). Her random urine chloride level greater than 20 mmol/L was indicative of a predominantly chloride-resistant metabolic alkalosis. In the setting of the patient’s hypertension associated with metabolic alkalosis and tendency to hypokalemia, further workup was obtained after her serum potassium level was repleted to 3.8 mEq/L, to rule out secondary causes of hypertension. Laboratory workup showed a serum renin level of 0.2 ng/mL/h (reference: 0.25–5.82 ng/mL) with normal serum aldosterone level of 3.6 ng/dL supine (Ref: if supine 3–16 ng/dl, upright less or equal to 28 ng/dl) and AM cortisol level of 13.4 mcg/dL (Ref at 7–9 am: 4.6–23.4 mcg/dL). A computed tomography scan of her abdomen was obtained and showed normal-appearing adrenal glands. Further, renal vascular ultrasound was unremarkable with no evidence of renal artery stenosis.

**Table 1:** Laboratory results over time

Labs	Time Point 1: Initial labs on presentation	Time Point 2: 3 days after stopping baking soda	Time Pont 3: Following resumption of baking soda us	Time Point 4: 3 days after re-stopping baking soda	Reference range
**Sodium**	146	140	145	141	135–148 mmol/L
**Potassium**	3.6	3.8	3.0	4.0	3.5–5.1 mmol/L
**Chloride**	95	98	100	102	96–109 mmol/L
**Bicarbonate**	44	34	39	32	21–31 mmol/L
**Urea Nitrogen**	8	9	9	6	7–22 mg/dL
**Creatinine**	1.37 (121.11)	1.55 (137.02)	1.40 (123.7)	1.40 (123.7)	0.6–1.3 mg/dL (53–114.9 umol/L)
**Glomerular Filtration Rate (eGFR)**	45	40	44	44	> 60 45 mL/min/1.73 sqm
**Glucose**	103	91	91	83	71–99 mg/dL
**Calcium**	9.3	9.1	8.9	8.8	8.4–10.5 mg/dL
**Albumin**	3.7	3.9	3.7	3.7	3.5–5.5 g/dL
**Magnesium**	2.0				1.7–2.2 mg/dL
**Arterial Blood Gas**					
**pH**	7.5				7.35–7.45
**PaCO2**	53.0 (7.06 kPa)				35–45 mmHg (4.67–6 kPa)
**HCO3**	41				22–26 mEq/L

**Fig. 1:**
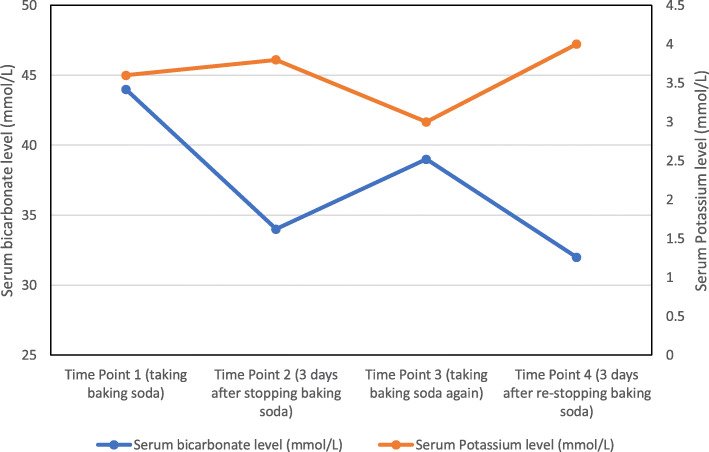
Serum Bicarbonate and serum potassium trend during the course of evaluation/management

Following initial extensive evaluation, the patient’s medications were again reviewed at a subsequent visit, this time with her daughter present. Upon questioning specifically on the use of baking soda, her daughter reported that the patient had been brushing her teeth with this substance at least three times a day for the prior 6 months. The patient reported that her friend recommended it for her to improve her teeth hygiene. Upon discontinuation of this practice, repeat labs 3 days later showed significant improvement in her serum bicarbonate level to 34 mmol/L, with a serum potassium level of 3.8 mmol/L (Table 1). However, the patient restarted brushing her teeth with baking soda and follow up labs showed again a higher serum bicarbonate of 39 mmol/L and a serum potassium of 3.0 mmol/L (Table 1). Again, following cessation of this practice, her repeat serum bicarbonate improved to 32 mmol/L with a serum potassium level of 4.0 mmol/L (Table 1 and Fig. 1). Her mild hypochloremia at presentation was most likely due to urine chloride loses (urine chloride was 48 mmol/L), precipitated by volume expansion due to chronic sodium bicarbonate use.

## Discussion and conclusions

This patient developed metabolic alkalosis due to exogenous alkali exposure from baking soda as a toothpaste additive, which to our knowledge has not been previously reported. Sodium bicarbonate toxicity in patients with CKD has been attributed to the impaired ability to excrete alkali loads [3]. This patient’s CKD placed her at a higher risk for toxicity. It is noted that the patient was on a low dose of calcium carbonate which provided further bases but did not explain the severe alkalosis by itself. In addition, our patient’s serum calcium level was normal. The fact that her alkalosis improved after cessation of baking soda on two separate occasions confirmed that this was the main source of her acid base derangement. It is conceivable that some people, for example those with some degree of cognitive impartment, may have small unintentional ingestion of toothpaste, which might have led to gastrointestinal absorption of baking soda in this patient’s case. One teaspoon of baking soda provides approximately 59 m-equivalents (mEq) of bicarbonate, while one 650-mg tablet of sodium bicarbonate provides only 7.7 mEq of bicarbonate [3, 4]. This explains the higher risk of alkalosis with a product that is widely available over the counter to all the population. Sodium bicarbonate toxicity is mostly related with its use as antacid and in smaller proportions as a strategy to cause false negative illicit drug tests and to treat UTIs [1–3]. Nonetheless, physicians should be aware of this common but underreported use of toxicity related to baking soda exposure.

## Data Availability

Not applicable.

## Abbreviations

mEq: milli-equivalents
CKD: Chronic Kidney Disease
eGFR: estimated Glomerular Filtration Rate
BMI: Body mass index
UTIs: Urinary Tract Infections
